# Association between magnesium and vitamin D status in adults with high prevalence of vitamin D deficiency and insufficiency

**DOI:** 10.1007/s00394-024-03559-9

**Published:** 2024-12-16

**Authors:** Armin Zittermann, Sieglinde Zelzer, Markus Herrmann, Marcus Kleber, Winfried Maerz, Sefan Pilz

**Affiliations:** 1 Clinic for Thoracic and Cardiovascular Surgery, Herz-und Diabeteszentrum NRW, Georgstr. 11, D-32545 Bad Oeynhausen, Germany; 2 Clinical Institute of Medical and Chemical Laboratory Diagnostics, Medical University of Graz, Graz, 8036 Austria; 3 Vth Department of Medicine (Nephrology, Hypertensiology, Rheumatology, Endocrinology, Diabetology, Lipidology), Medical Faculty Mannheim, University of Heidelberg, 68167 Mannheim, Germany; 4 SYNLAB MVZ Humangenetik Mannheim, 68163 Mannheim, Germany; 5 Division of Endocrinology and Diabetology, Department of Internal Medicine, Medical University of Graz, Graz, 8036 Austria; 6 SYNLAB Holding, Deutschland GmbH, 68159 Mannheim, Augsburg, Germany

**Keywords:** 25-hydroxyvitamin D, Calcitriol, 24,25(OH)_2_D_3_, Hypomagnesemia, Vitamin D metabolite ratio

## Abstract

**Purpose:**

It has been assumed that magnesium (Mg) status may interact with vitamin D status. We therefore aimed at investigating the association between Mg and vitamin D status in a large cohort of adult individuals with a high prevalence of deficient/insufficient vitamin D and Mg status.

**Methods:**

We used data from the Ludwigshafen Risk and Cardiovascular Health Study (*n* = 2,286) to analyze differences according to serum Mg status in circulating 25-hydroxyvitamin D [25(OH)D] (primary endpoint), 24,25-dihydroxyvitamin D_3_ [24,25(OH)_2_D_3_], vitamin D metabolite ratio and calcitriol, and odds ratios for deficient or insufficient 25(OH)D (secondary endpoints). We performed unadjusted and risk score (RS) adjusted and matched analyses.

**Results:**

Of the study cohort (average age > 60 years), one third was 25(OH)D deficient (< 12 ng/mL), one third 25(OH)D insufficient (12 to < 20 ng/mL), about 10% Mg deficient (< 0.75 mmol/L) and additional 40% potentially Mg deficient (0.75 to 0.85 mmol/L). In adjusted/matched analyses, 25(OH)D was only non-significantly lower in Mg deficient or insufficient groups versus their respective control group (*P* > 0.05). Only the RS-adjusted, but not the RS-matched odds ratio of 25(OH)D deficiency was significantly lower for the group with adequate versus deficient/potentially deficient Mg status (0.83; 95%CI: 0.69–0.99), and only the RS-matched, but not the RS-adjusted odds ratio of 25(OH)D insufficiency was significantly lower for non-deficient versus deficient Mg status (0.69; 95%CI: 0.48–0.99). Other adjusted or matched secondary endpoints did not differ significantly between subgroups of Mg status.

**Conclusions:**

Our data indicate only little effect between Mg and vitamin D status in adults with high prevalence of vitamin D deficiency and insufficiency.

**Supplementary Information:**

The online version contains supplementary material available at 10.1007/s00394-024-03559-9.

## Introduction

Vitamin D deficiency is considered to be a panacea [1]. Adequate vitamin D status, defined as circulating 25-hydroxyvitamin D [25(OH)] greater than 20 ng/mL (multiply by 2.496 to convert to nmol/L), is present in less than 50% of the world population [2]. In Europe, standardized values of circulating 25(OH)D showed that 13.0% of the 55,844 European individuals studied had deficient (< 12 ng/mL) and another 40.4% at least insufficient (12 to ≤ 20 ng/mL) concentrations [3].

Insufficient magnesium (Mg) supply is an issue as well. Epidemiologic studies in Europe, North America and other countries show that the Mg intake is less than 30–50% of the recommended daily allowance (RDA) of 420 mg/day for men and 320 mg/day for women [4]. In high-income countries, the prevalence of subclinical magnesium deficiency based on serum Mg levels < 0.8 mmol/L is estimated to be around 10–30% [5].

Mg is an inorganic cofactor required for many enzymatic reactions, among them the conversion of vitamin D into 25(OH)D by a hepatic 25-hydroxlase (CYP2R1) and a subsequent synthesis of 1,25-dihydroxyvitamin D (designated calcitriol) by a renal 1α-hydroxylase (CYP27B1). The first step of 25(OH)D inactivation into 24,25-dihydroxyvitamin D (24,25(OH)_2_D) by a 24-hydroxylase (CYP24A1) is also Mg-dependent [1].

It has been assumed that an inadequate Mg supply may adversely affect circulating vitamin D metabolite concentrations [6]. This assumption is supported by two case reports demonstrating that treatment with Mg supplements was more effective than with vitamin D supplements in healing rickets [7]. In addition, there was a reduced risk of deficient or insufficient (< 20 ng/mL) concentrations of circulating 25(OH)D in two large data sets from the National Health and Nutrition Examination Surveys III [8] at high Mg intake. Likewise, a recent randomized controlled trial (RCT) in 180 patients reported that Mg supplementation significantly influences vitamin D metabolism, dependent on the vitamin D status at baseline: serum concentrations of 25(OH)D increased with Mg supplementation only when baseline 25(OH)D were < 30 ng/mL but decreased when baseline 25(OH)D was higher (from 30 to 50 ng/mL) [9], indicating that optimal magnesium status may be important for optimizing 25(OH)D status. Mg treatment also significantly influenced 24,25(OH)_2_D_3_ concentrations when baseline 25(OH)D concentration was 50 ng/mL but not 30 ng/mL [9]. Furthermore, Mg supplementation resulted in a significant increase in circulating 25(OH)D in two others, small, RCTs [10, 11], but in both trials the 25(OH)D increase was not significant versus the change in the placebo group.

Some reviews have already concluded that for normal vitamin D metabolism and activity, an optimal Mg status is required [12] and adequate Mg supplementation should be considered as an important aspect of vitamin D therapy [13]. However, based on the aforementioned scientific data we hypothesized that in general populations an association of Mg status with vitamin D status, if any, is weak at best. To test this hypothesis, we aimed to investigate the potential interaction of serum Mg concentrations with vitamin D metabolite concentrations in a post-hoc analysis of a large cohort of adults at high risk of cardiovascular disease (CVD), since two third of these individuals had deficient/insufficient vitamin D status [14] and it can also be assumed from published data on serum Mg that a substantial percentage of these individuals had subclinical Mg deficiency [15].

## Methods

### Patients and methods

For this retrospective, cross-sectional study, we used data from The Ludwigshafen Risk and Cardiovascular Health Study (LURIC). LURIC is an academic collaboration of the Ludwigshafen Heart Centre with several academic partners (located at the universities of Freiburg, Ulm and Düsseldorf, Germany, and the Centre Nationale de Genotypage, Evry, France) to assess classical and new parameters of CVD risk [16]. The main site is the Ludwigshafen Heart Centre, a tertiary care center, in southwest Germany (geographic latitude: 49.5°N). LURIC enrolled a total of 3316 Caucasian patients. For the present data analysis, we assessed the following baseline characteristics: sex, age, body mass index, season of blood sampling, smoking, exercise (scale of 1 [low] to 11 [high]), estimated glomerular filtration rate (eGFR, calculated according to the Chronic Kidney Disease Epidemiology Collaboration (CKD-EPI) formula [17]), diagnoses, such as diabetes mellitus, hypertension, and atrial fibrillation, routine biochemical parameters, such as bilirubin, sodium, calcium, phosphate, parathyroid hormone (PTH), C-reactive protein (CRP), and cortisol, and medications, such as beta-blockers, angiotensin converting enzyme-inhibitors/angiotensin 1 receptor-blockers, glucocorticoids, statins, digitoxin, diuretics, aspirin, and antibiotics. In addition, more recently measured vitamin D metabolites 25(OH)D and 24,25(OH)_2_D_3_ [18] from deeply frozen, stored serum samples were used. Since Mg concentrations < 0.85 mmol/L were classified as deficient/potentially deficient and values > 0.85 mmol/L as adequate [19], we divided the study cohort in two subgroups (deficient/potentially deficient: < 0.85; adequate: ≥ 0.85 mmol/L). The cutoffs of serum Mg are based on associations of risk factors for chronic disease such as glucose intolerance, inflammation, and elevated blood pressure with serum Mg concentrations in the range of 0.75–0.85 mmol/L [19]. In sensitivity analysis, we chose deficient Mg concentrations (< 0.75 mmol/L) and non-deficient Mg concentrations (≥ 0.75 mmol/L) as cutoffs for dividing the study cohort in two subgroups. Patients were classified as vitamin D deficient and insufficient if 25OHD levels were < 12 ng/mL and between 12 and 20 ng/mL, respectively [9]. The LURIC study was approved by the institutional review board of the ethics committee of the Landesärztekammer Rheinland-Pfalz (No. 1997 − 203) and was performed in adherence to the principles of the Declaration of Helsinki. All subjects gave written informed consent.

## Biochemical analyses

Serum calcium, sodium, Mg, phosphate, creatinine, bilirubin, CRP, cortisol, and intact PTH were measured using automated routine methods. Test procedures, coefficients of variation (CVs), and reference values are listed in Supplemental Table 1. Regarding these routinely measured biochemical analytes, the laboratory participated successfully in legally required external quality controls. Of the 3,316 samples, 25(OH)D (sum of 25(OH)D_2_ and 25(OH)_3_) and 24,25(OH)_2_D_3_ were measured in a subset of 2,477 samples using liquid chromatography tandem mass spectrometry (LC-MS/MS), as previously described [20]. For both vitamin D metabolites, intra- and inter-assay coefficients of variation (CVs) were 9% and 12%, respectively. In addition, the vitamin D metabolite ratio (VMR, in %), i.e. 24,25(OH)_2_D divided by 25(OH)D, was determined [20]. The LC-MS/MS method of quantifying 25(OH)D and 24,25(OH)_2_D has performed satisfactorily in the Vitamin D External Quality Assessment Scheme (DEQAS) [10]. Levels of calcitriol were measured by radioimmunoassay from Nichols Institute Diagnostika GmbH (Bad Nauheim, Germany) with intra- and inter-assay CVs both < 10%. The reference range was, according to the manufacturer, 18–72 pg/mL.

## Endpoints

Primary endpoint was circulating 25(OH)D. Secondary endpoints were the relative risk of deficient or insufficient 25(OH)D, as well as circulating 24,25(OH)_2_D_3_, VMR, and calcitriol concentrations.

## Statistics

We report categorical variables as number and percentage of observations. The chi-squared test was used to compare results of categorical variables between subgroups. Continuous baseline variables are presented as mean with standard deviation (SD). Continuous outcome parameters are presented as mean with 95% confidence interval (CI) and categorical outcome parameters are given as odds ratio (OR) with 95% confidence interval (CI). No data imputation was performed for missing values.

We used different statistical approaches to confirm agreement regarding the association of Mg status with vitamin D metabolites. We first conducted unadjusted ANCOVA (analysis of covariance) by Mg status and performed unadjusted binary logistic regression analysis to assess the OR of deficient or insufficient circulating 25(OH)D by Mg status. In a second step, similar to propensity score modelled analysis [21], we generated a score of baseline characteristics, including CVD parameters [22]. We designated this score “risk score (RS)” of being in the subgroup of deficient/potentially deficient Mg concentrations. We performed RS-adjustment and matching to avoid biased results. The use of a single score also prevented overparameterizing the model.

The RS derivation model used all potential confounders listed in Table 1 and was constructed with multivariable logistic regression, with Mg status as the binominal dependent variable and the parameters of Table 1 as predictor variables. They were selected because of associations with study cohort and/or potential clinically relevant associations with Mg status and/or vitamin D metabolism. The RS was placed in the main covariate-adjusted models as an independent variable along with Mg status (deficient/potentially deficient, adequate). With respect to the covariate-adjusted analysis of the primary endpoint, the Levene test was used to check equality of variances between groups. Cases with a leverage above 0.2 were considered as outliers. We also performed bootstrapping by resampling the prediction population 1000 times with replacement to allow for a more robust estimation of the parameters that might otherwise be biased by a lack of homogeneity of variance. Similar to the approach of PS-matching, we performed RS-matching. Matching was performed using a 1:1 ratio with the logit-transformed RS. For this, an optimal-matching algorithm with a caliper width of 0.1 standard deviation (SD) from the linear predictor was used. Percentages and means with SD were both used to calculate standardized mean difference (SMD). Balance of risk factors was judged by standardized mean differences (SMD). The balance is considered to be satisfactory when the SMD is less than 10% [23].

**Table 1:** Characteristics of unmatched and matched study participants by serum Mg status

	Unmatched	patients (*n* = 2,286)		Matched	patients (*n* = 1,860)	
Parameter	Deficient/potentially deficient Mg status *n* = 1164	Adequate Mg status *n* = 1122	SMD %	Deficient/potentially deficient Mg status *n* = 930	Adequate Mg status *n* = 930	SMD%
Age (years)	62.2 ± 10.6	62.7 ± 10.5	-4.7	62.9 ± 10.8	63.0 ± 10.5	-0.9
Females	335 (28.8)	386 (34.4)	-16.4	291 (31.3)	291 (31.3)	0.0
Body Mass Index (kg/m^2^)	27.4 ± 4.0	27.5 ± 4.2	-2.4	27.4 ± 3.9	27.5 ± 4.2	-2.5
Season of Blood Sampling						
Winter	224 (19.3)	333 (29.7)	-31.7	213 (22.9)	208 (22.4)	1.7
Spring	185 (15.9)	242 (21.6)	-19.2	284 (30.5)	201 (21.6)	-12.8
Summer	356 (30.6)	294 (26.2)	13.9	284 (30.5)	271 (29.1)	4.3
Fall	399 (34.3)	253 (22.5)	39.1	267 (28.7)	250 (26.9)	5.6
Smoking	766 (65.8)	690 (61.5)	12.4	597 (64.2)	582 (62.6)	4.6
Exercise (scale)	5.9 ± 1.8	5.9 ± 1.7	0.0	5.9 ± 1.8	5.9 ± 1.8	0.0
eGFR (ml/min/1.73 m^2^)	63.9 ± 15.2	67.8 ± 15.1	-25.7	65.5 ± 14.4	66.0 ± 14.3	-3.5
Diagnosis						
Diabetes mellitus	188 (16.2)	226 (20.1)	-13.4	154 (16.6)	171 (18.4)	-6.4
Hypertension	666 (57.2)	656 (58.5)	-3.7	536 (57.2)	532 (57.6)	-1.1
Atrial Fibrillation	168 (14.4)	176 (15.7)	-4.9	143 (15.4)	141 (15.2)	0.8
Biochemical Parameters						
Bilirubin (mg/dL)	0.65 ± 0.41	0.63 ± 0.36	5.2	0.64 ± 0.37	0.64 ± 0.38	0.0
Sodium (mmol/L)	141 ± 3	141 ± 3	0.0	141 ± 3	141 ± 3	0.0
Calcium (mmol/L)	2.33 ± 0.11	2.32 ± 0.10	9.5	2.33 ± 0.11	2.33 ± 0.10	0.0
Phosphate (mmol/L)	1.14 ± 0.19	1.14 ± 0.18	0.0	1.14 ± 0.18	1.13 ± 0.18	5.6
PTH (pg/mL)	36.3 ± 41.0	32.1 ± 15.9	13.5	32.3 ± 16.9	32.8 ± 16.6	-3.6
C-reactive protein (mg/dL)	1.02 ± 2.04	0.92 ± 1.78	5.2	0.97 ± 1.85	0.96 ± 1.88	0.5
Cortisol (µg/dL)	23.1 ± 9.6	21.9 ± 6.8	14.4	22.6 ± 7.1	22.3 ± 6.8	4.3
Medications						
Beta-Blockers	744 (63.9)	700 (62.4)	4.3	584 (63.3)	589 (62.8)	1.5
ACE-inhibitors/AT-blockers	644 (57.4)	678 (58.2)	-2.3	547 (58.8)	540 (58.1)	2.0
Glucocorticoids	28 (2.4)	22 (2.0)	3.3	18 (1.7)	16 (1.9)	-1.7
Statins	591 (50.8)	494 (44.0)	19.2	437 (48.8)	454 (47.0)	5.0
Digitoxin	169 (14.5)	179 (16.0)	-5.6	149 (14.9)	139 (16.0)	-4.1
Diuretics	314 (29.5)	343 (28.0)	4.6	264 (28.4)	268 (28.8)	-1.2
Aspirin	840 (72.2)	791 (70.5)	5.2	658 (70.8)	665 (71.5)	-2.2
Antibiotics	22 (1.9)	17 (1.5)	3.6	16 (1.7)	14 (1.5)	1.8

Abbreviations: eGFR, estimated glomerular filtration rate; PTH, parathyroid hormone; ACE, angiotensin converting enzyme; AT, angiotensin; SMD, standardized mean difference

In sensitivity analysis, we conducted RS-adjusted and RS-matched analysis according to deficient versus non-deficient Mg status, i.e. serum Mg < 0.75 and ≥ 0.75 mmol/l [19]. Based on results by Deng et al. regarding the 25(OH)D_3_ increase in the group with baseline 25(OH)D of 30 ng/ml by Mg supplementation [8], we calculated that a total of 1054 patients (527 per group) has to enter our study when assuming a difference between study groups of + 2.0 ng/ml and an SD of 10 ng/ml. Then, the probability is 90% that the study will detect this difference at a two-sided 0.05 significance level.

The software package IBM SPSS, version 24 (IBM Corp, Armonk, NY, USA) was used for the statistical analyses, with the exception of bootstrapping, which was performed with IBM SPSS version 27. Additionally, the PSMATCHING3 R Extension 4 command (Version 2.15.3, R Core Foundation, Austria, Vienna) was added as an SPSS extension bundle under the SPE file format to be able to run this extra program feature in SPSS version 24.

## Results

### Baseline characteristics

Of the 2,477 patients with available LC MS/MS measurements of 25(OH)D and 24,25(OH)_2_D_3_, 191 had to be excluded because of missing covariate data, leaving 2,286 patients for final data analysis. Mean age of the study cohort was 62.5 years (SD: 10.6 years), two third of patients were male and two third were smokers. Physical activity was on average moderate (Table 1). Only 41 patients (1.8%) had no chronic diseases such as CVDs and/or diabetes mellitus. Blood samples were collected during all seasons. Circulating 25(OH)D and Mg concentrations ranged from 1.8 to 182 ng/mL and 0.4 to 1.58 mmol/L, respectively. Of the study cohort, one third was vitamin D deficient, one additional third vitamin D insufficient, about 10% magnesium deficient, and additional 40% potentially magnesium deficient, with some variations according to demographic, anthropometric, and clinical data (Supplemental Table 2). Double-deficiency of vitamin D and Mg was present in 4.1% of the study cohort and additional 29.3% were at least vitamin D insufficient *and* potentially Mg deficient.

**Fig. 1:**
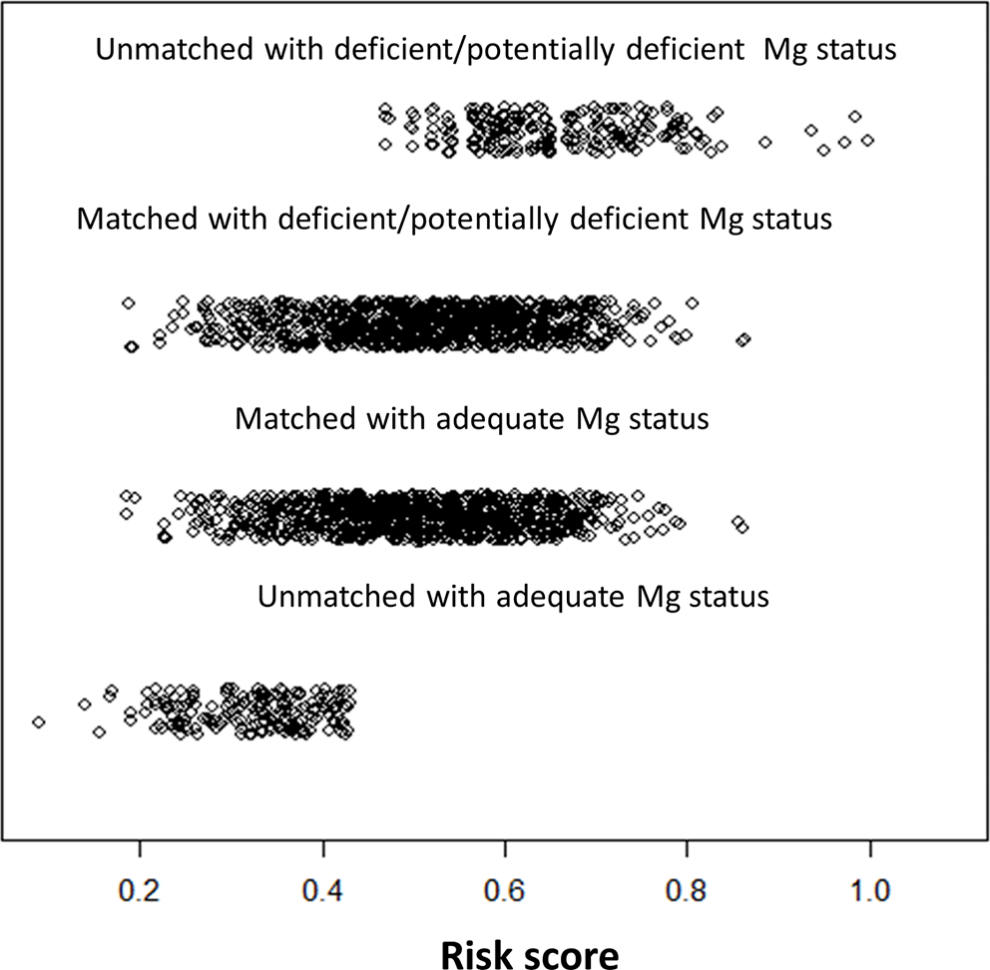
Risk scores in matched and unmatched patients with deficient/potentially deficient or adequate Mg status

In the groups with deficient/potentially deficient and adequate Mg status, average serum Mg was 0.78 and 0.93 mmol/L, respectively. Table 1 presents characteristics of patients by serum Mg status in the entire cohort and in the RS-matched groups. Briefly, season of blood drawing, sex distribution, kidney function, prevalence of diabetes mellitus, and statin use differed substantially between unmatched study groups. The RS ranged from a low of 0.0892 to a high of 0.9983 (Fig. 1).

## Primary endpoint

In the entire study cohort, circulating 25(OH)D concentrations were on average 17.2 ng/mL. Unadjusted circulating 25(OH)D was similar between study groups (Table 2). In the RS-adjusted ANCOVA, results of circulating 25(OH)D also did not differ statistically significantly between study groups (Table 2). The assumption of homogeneity of variances was found to be satisfied, as assessed by Levene’s test (*P* = 0.58). In addition, no outliers were present (all leverages < 0.2). RS-matching reduced the SMD in preoperative covariates between the study groups substantially. In the RS-matched groups, all standardized differences were < 10%, with the exception of slightly less blood drawings in spring in the deficient/potentially deficient Mg group than in the adequate Mg group (Table 1). The RS-matched analysis (*n* = 930 pairs) revealed no statistically significant difference in circulating 25(OH)D between study groups as well (Table 2). Bootstrapping did not change results substantially (Supplemental Table 3).

**Table 2:** Differences in vitamin D metabolite concentration and odds ratios of 25-hydroxyvitamin D deficiency or insufficiency between deficient/potentially deficient and adequate Mg status

Parameter	Deficient/potentially deficient Mg status	Adequate Mg status	Difference or odds ratios with 95%CI	*P*-value
*Primary endpoint*				
*25(OH)D (ng/mL; mean*,*95%CI)*				
Unadjusted	17.4 (16.8 to 17.9)	17.1 (16.5 to 17.7)	-0.27 (-1.07 to 0.54)	0.52
RS-adjusted	17.0 (16.4 to 17.5)	17.5 (17.0 to 18.1)	0.57 (-0.25 to 1.40)	0.18
RS-matched	17.3 (17.0 to 18.3)	17.6 (16.6 to 17.9)	0.31 (-0.46 to 1.37)	0.51
*Secondary Endpoints*				
*Deficient 25(OH)D (%*,* OR*,*95%CI)*				
Unadjusted	33.3	33.2	1.00 (0.84 to 1.19)	0.96
RS-adjusted	-	-	0.83 (0.69 to 0.99)	0.043
RS-matched	35.2	31.7	0.86 (0.71 to 1.04)	0.12
*Insufficient 25(OH)D (%*,* OR*,*95%CI)*				
Unadjusted	65.8	68.1	1.11 (0.93 to 1.32)	0.25
RS-adjusted	-	-	0.93 (0.77 to 1.11)	0.41
RS-matched	66.1	65.81	0.99 (0.81 to 1.19)	0.88
*24*,*25(OH)*_2_*D (ng/mL; mean*,*95%CI)*				
Unadjusted	2.96 (2.81 to 3.12)	2.85 (2.69 to 3.01)	-0.12 (-0.34 to 0.11)	0.31
RS-adjusted	2.86 (2.70 to 3.02)	2.96 (2.80 to 3.12)	0.10 (-0.13 to 0.34)	0.38
RS-matched	2.96 (2.78 to 3.15)	2.99 (2.81 to 3.18)	0.03 (-0.27 to 0.29)	0.83
*Vitamin D metabolite ratio (%*,* mean*,*95%CI)*				
Unadjusted	6.31 (6.16 to 6.48)	6.10 (5.94 to 6.27)	-0.22 (-0.45 to 0.01)	0.06
RS-adjusted	6.22 (6.05 to 6.38)	6.21 (6.05 to 6.38)	-0.01 (-0.24 to 0.23)	0.98
RS-matched	6.24 (6.06 to 6.42)	6.30 (6.11 to 6.48)	0.05 (-0.20 to 0.31)	0.68
*Calcitriol (pg/mL; mean*,*95%CI)*				
Unadjusted	34.2 (33.4 to 35.0)	35.1 (34.4 to 36.0)	0.97 (-0.15 to 2.09)	0.09
RS-adjusted	34.1 (33.4 to 35.0)	35.2 (34.4 to 36.0)	1.06 (-0.10 to 2.22)	0.07
RS-matched	34.6 (33.8 to 35.5)	35.5 (34.6 to 36.4)	0.85 (-0.38 to 2.09)	0.18

Abbreviations: 25(OH)D, 25-hydroxyvitamin D; CI, confidence interval; RS, risk score; OR, odds ratio; *24*,25(OH)_2_D, 24,25-dihydroxyvitamin D

## Secondary endpoints

The OR of deficient 25(OH)D concentrations differed significantly between study groups, at least in the RS-adjusted analysis, whereas the OR of insufficient 25(OH)D was similar between groups (Table 2). RS-adjusted or RS-matched secondary endpoints such as 24,25(OH)_2_D_3_, VMR, and calcitriol did not differ significantly between study groups (Table 2).

### Sensitivity analyses

With respect to the sensitivity analyses, baseline characteristics of the two study groups are presented in Supplemental Table 4 according to RS-matched groups (*n* = 292 pairs). All SMDs in preoperative covariates were below 10% (Supplemental Table 4). The homogeneity of variances was found to be satisfied (Levene’s test *P* = 0.51) and no outliers were present (all leverages < 0.2) in the RS-adjusted analysis. The RS scores are depicted in Supplemental Fig. 1 by study group. Regarding the primary endpoint, at least in the RS-matched analysis circulating 25(OH)D was non-significantly (*P* = 0.06) 1.5 ng/mL higher in the group with non-deficient Mg status than with deficient Mg status (Table 3). In addition, the OR of insufficient circulating 25(OH)D was significantly lower in RS-matched analysis in the group with non-deficient than deficient Mg status. Other secondary endpoints did not differ significantly between the group with deficient and non-deficient Mg status (Table 3). Bootstrapping did not change results of the primary endpoint substantially (Supplemental Table 3).

**Table 3:** Differences in vitamin D metabolite concentration and odds ratios of 25-hydroxyvitamin D deficiency or insufficiency between deficient and non-deficient Mg status

Parameter	Deficient Mg status	Non-deficient Mg status	Difference or odds ratios with 95%CI	*P*-value
*Primary endpoint*				
*25(OH)D (ng/mL; mean*,*95%CI)*				
Unadjusted	17.0 (15.9 to 18.2)	17.3 (16.8 to 17.7)	0.23 (-0.98 to 1.44)	0.71
RS-adjusted	17.1 (16.6 to 17.5)	17.8 (16.6 to 19.1)	0.79 (-0.54 to 2.10)	0.25
RS-matched	5.7 (14.6 to 16.7)	17.1 (16.1 to 18.2)	1.54 (-0.03 to 2.91)	0.06
*Secondary Endpoints*				
*Deficient 25(OH)D (%*,* OR*,*95%CI)*				
Unadjusted	32.9	35.6	1.13 (0.87 to 1.45)	0.37
RS-adjusted	-	-	0.83 (0.63 to 1.09)	0.18
RS-matched	39.4	35.3	0.84 (0.60 to 1.17)	0.31
*Insufficient 25(OH)D (%*,* OR*,*95%CI)*				
Unadjusted	67.5	66.9	0.97 (0.75 to 1.27)	0.84
RS-adjusted	-	-	0.76 (0.58 to 1.01)	0.06
RS-matched	74.5	67.1	0.69 (0.48 to 0.99)	0.046
*24*,*25(OH)*_2_*D (ng/mL; mean*,*95%CI)*				
Unadjusted	2.73 (2.81 to 3.05)	2.93 (2.42 to 3.05)	0.20 (-0.14 to 0.54)	0.25
RS-adjusted	2.92 (2.57 to 3.27)	2.88 (2.76 to 3.00)	-0.04 (-0.41 to 0.33)	0.84
RS-matched	2.75 (2.47 to 3.03)	2.55 (2.27 to 2.83)	-0.20 (-0.59 to 0.20)	0.33
*Vitamin D metabolite ratio (%*,* mean*,*95%CI)*				
Unadjusted	5.96 (5.64 to 6.79)	6.25 (6.13 to 6.37)	0.29 (-0.09 to 0.63)	0.09
RS-adjusted	6.26 (5.94 to 6.58)	6.21 (6.09 to 6.33)	-0.06 (-0.40 to 0.29)	0.76
RS-matched	5.97 (5.67 to 6.27)	5.97 (5.67 to 6.27)	0.00 (–0.43 to 0.43)	> 0.99
*Calcitriol (pg/mL; mean*,*95%CI)*				
Unadjusted	34.7 (33.1 to 36.3)	34.7 (34.1 to 35.3)	-0.05 (-1.72 to 1.62)	0.95
RS-adjusted	35.1 (33.3 to 36.4)	34.6 (34.0 to 35.2)	-0.46 (-2.32 to 1.40)	0.63
RS-matched	34.8 (33.3 to 36.4)	33.6 (32.0 to 35.1)	-1.27 (-3.42 to 0.87)	0.24

Abbreviations: 25(OH)D, 25-hydroxyvitamin D; CI, confidence interval; RS, risk score; OR, odds ratio; *24*,25(OH)_2_D, 24,25-dihydroxyvitamin D

## Discussion

In the present investigation, we aimed to investigate the potential interaction of serum Mg concentrations with vitamin D metabolite concentrations in a series of patients undergoing coronary angiography. We found no statistically significant difference in circulating 25(OH)D between study participants with deficient/potentially deficient or adequate Mg status. Likewise, secondary endpoints such as circulating 24,24(OH)_2_D_3_, VMR, and calcitriol concentrations did not differ significantly by Mg status. The only exceptions were significant lower odds ratios of deficient/insufficient vitamin D status in those with deficient/potentially deficient Mg status in some, but not all RS-adjusted or matched analyses.

This study is important because, to the best of our knowledge, it is the first analysis investigating in a large cohort of patients the association of serum Mg status with concentrations of various vitamin D metabolites, including the most active, hormonal form of vitamin D, calcitriol. Moreover, the prevalence of deficient/insufficient vitamin D and Mg status was high, so that the cohort was well suitable for the present study. In all RS-adjusted and matched analyses, circulating 25(OH)D was only slightly (0.31 to 1.54 pg/mL) and non-significantly lower in subgroups with deficient or potentially deficient Mg status than in their respective controls. In two small trials [10, 11], the mean increase in total 25(OH)D was about 3 ng/mL higher in the Mg supplemented groups than in the placebo groups, but the increases did not reach statistical significance. Even in patients with very low serum Mg concentrations (mean: 0.41 mmol/L) and mean 25(OH)D of 13.2 ng/mL, parenteral Mg therapy did not increase circulating 25(OH)D significantly [24]. In another trial [25], vitamin D plus Mg supplementation resulted in a significant increase versus vitamin D plus placebo supplementation only in the subgroup with initial 25(OH)D concentrations < 20 ng/mL, but not in the entire study cohort. A further trial reported that Mg supplementation increased the 25(OH)D_3_ concentration only when baseline 25(OH)D concentrations were close to 30 ng/mL [9]. Data of NHANES III [8] indicated a lower OR for insufficient or deficient 25(OH)D status in the highest quartile of Mg intake (> 420 mg/d) versus the lowest quartile of Mg intake (< 225 mg/d). Some of our RS-adjusted or matched results are in line with these earlier findings, whereas other analyses revealed non-significant results. Overall, the inconsistent data support the assumption of little effect of Mg status on circulating 25(OH)D.

In line with our results, others reported no significant effect of Mg supplementation on 24,25(OH)_2_D_3_ and calcitriol at initial 25(OH)D concentrations < 20 ng/mL [9]. Likewise, in patients with mean serum Mg concentrations of 0.41 mmol/L [25], parenteral Mg therapy did not increase calcitriol significantly. Probably, the effect of Mg deficiency is limited to patients with severe Mg deficiency, since in two case reports on rickets in connection with hypomagnesemia [7] and successful treatment of the bone disease with Mg administration, the serum Mg concentration was 0.21 and 0.30 mmol/l, which is extremely low.

Our study has both strengths and limitations. Strengths include (i) the rigorous statistical approaches and the large number of covariates used to consider potential confounding, (ii) the inclusion of almost all patients with deficient Mg status in the RS-matched sensitivity analysis, (iii) the large number of study participants, (iv) the use of serum Mg concentrations to assess Mg status, and (v) the measurement of different vitamin D metabolites. One limitation of our study is its cross-sectional design, which is prone to reverse causation bias and unexplained confounding. Another limitation is that no dietary intake data and no urinary output data were available. However, dietary Mg intake is prone to over- or underreporting and differences in intestinal Mg uptake. Daily urinary Mg excretion is potentially useful [19], but is rarely assessed in large cohort studies. Furthermore, matching of study groups is usually performed instead of randomization, i.e. if different surgical approaches are compared, and effects may be interpreted as causal. Since no randomization is generally possible according to serum Mg concentrations, the problem of unexplained confounding remains, even after matching of study groups. Nevertheless, it is also noteworthy that at least for the primary endpoint the different approaches we used in our statistical analyses achieved almost similar results. Finally, we were unable to analyze the association between Mg status and vitamin D status in a control group of apparently healthy individuals, as this group was very small (*n* = 41) in our study cohort. In this small group, a potential Mg effect on the primary endpoint would have had to be much higher than the 2 ng/ml calculated in 1054 patients to be statistically significant. However, we are not aware of any specific CVD or diabetes mellitus effects on the interaction of Mg on vitamin D metabolism. We can therefore reliably assume that the results of the present study can be generalized to the adult population as a whole.

## Conclusion

Our data indicate only little effect between Mg and vitamin D status in adults with high prevalence of vitamin D deficiency and insufficiency. However, in some, but not all statistical analyses, the RS-adjusted or matched OR of 25(OH)D deficiency/insufficiency was significantly lower for the groups with non-deficient/adequate versus deficient/potentially deficient Mg status. In future, more data from prospective studies using different methods of assessing Mg status such as dietary Mg intake, serum Mg concentrations, and urinary Mg excretion, are warranted. These studies should focus on individuals with deficient/insufficient vitamin D status, and should also include specific groups with an increased Mg need such as athletes and pregnant women as well as individuals with severe Mg deficiency, i.e. serum Mg concentration ≤ 0.40 mmol/L.

## Electronic supplementary material

Below is the link to the electronic supplementary material.

Supplementary Material 1
