# Supplementary material for: Association between magnesium and vitamin D status in adults with high prevalence of vitamin D deficiency and insufficiency
Source: Eur J Nutr. 2024 Dec 16;64(1):48. doi: 10.1007/s00394-024-03559-9 (PMC11649730; doi:10.1007/s00394-024-03559-9)
Supplement: Supplementary file 1 — Supplementary Material 1 [file 394_2024_3559_MOESM1_ESM.pdf]

Supplemental Material

Association of magnesium status with vitamin D status in adults with a high prevalence of vitamin D deficiency and insufficiency

Zittermann A, Zelzer S, Herrmann M, Kleber M, Maerz W, Pilz S

		**Page**
**Supplemental Figure 1:**	Risk scores in matched and unmatched patients with deficient or non-deficient Mg status	2
**Supplemental Table 1:**	Summary of methods and test kits used for laboratory analysis and their reference range, and intra- and inter-assay coefficients of variation	3
**Supplemental Table 2**:	Vitamin D and magnesium status in the entire study cohort and according to different demographic, anthropometric, and clinical parameters	4
**Supplemental Table 3**	Results of bootstrapping for the primary endpoint 25-hydroxyvitamin D using 1,000 samples with random replacement	5
**Supplemental Table 4:**	Characteristics of unmatched and matched study participants with deficient/non-deficient or adequate serum Mg status	6

Supplemental Figure 1: Risk scores in matched and unmatched patients with deficient or non-deficient Mg status

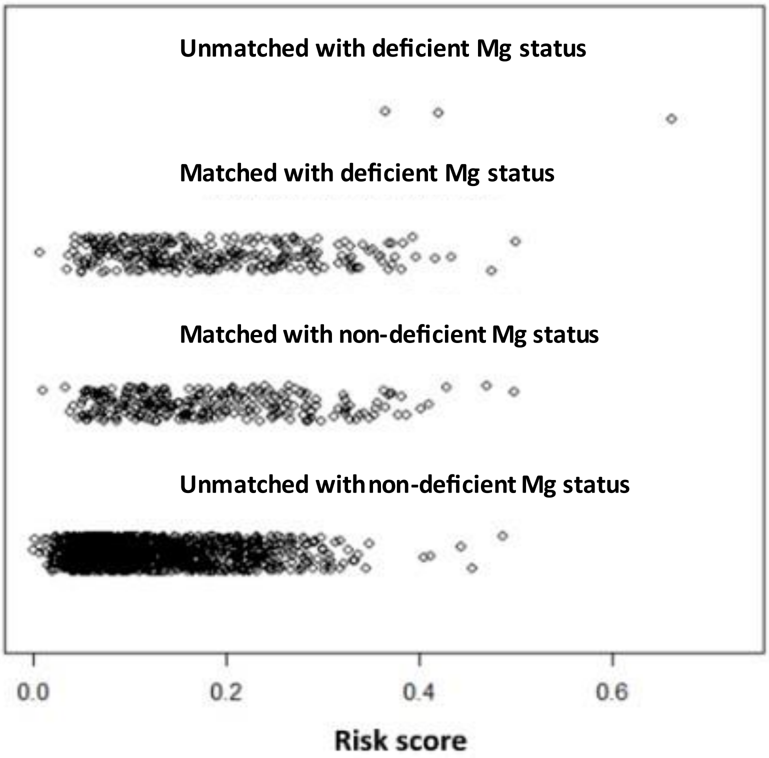

Supplemental Table 1: Summary of methods and test kits used for laboratory analysis and their reference range, and intra- and inter-assay coefficients of variation

Parameter	Method	Analytical system	Provider	Reference range	Intra- and inter-assay CV
Bilirubin, total	DPD method	BIL-T/Hitachi 717	Roche Mannheim, Germany	0.2 – 1.0 mg/dl	< 2.5% and <4%
Calcium	o-cresolphthalein-complex	CA/Hitachi 717	Roche Mannheim, Germany	2.1- 2.75 mmol/L	< 2.5% and < 4%
Cortisol	Solid-phase chemiluminescence enzyme immunoassay	Cortisol-Immulite®/ DPC Immulite autosampler	DPC Biermann GmbH Bad Nauheim, Germany	5-25 µg/dl	both < 10%
C-reactive protein	Immunoturbidimetric assay	CRP/Hitachi 911	Roche Mannheim, Germany	< 0.5 mg/dl	4.0% and 8.5%
Creatinine	Jaffé method (twin mode)	CREA/Hitachi 717	Roche Mannheim, Germany	< 1.2 mg/dl	<2.5% and < 4%
Intact PTH	Chemiluminescence enzyme immunoassay	iPTH/Elecsys 2010	Roche Diagnostics GmbH Mannheim, Germany	15-65 pg/mL	both < 10%
Phosphate, inorganic	Molybdate-reaction	PHOS/Hitachi 717	Roche Mannheim, Germany	0.97 – 1.62 mmol/L	<2.5% and < 4%
Sodium	Ion selective electrode (ISE)	ISE diluent, ISE Internal Reference and Reference Electrode Solution/Hitachi 717	Roche Diagnostics GmbH Mannheim, Germany	137 – 147 mmol/L	< 2.5% and < 4%
Magnesium	Colorimetric method	Cobas Mira Plus Roche autoanalyzer	Roche Diagnostics GmbH Mannheim, Germany	see Methods section	both < 4%

Supplemental Table 2: Vitamin D and magnesium status in the entire study cohort and according to different demographic, anthropometric, and clinical parameters

	Vitamin D deficient		Vitamin D insufficient	P-value	Magnesium deficient	Possibly magnesium deficient	P-value
Entire cohort (n,%)	780 (34.1)		736 (32.2)	-	272 (11.9)	905 (39.6)	-
Sex (n,%)				<0.001			0.008
Males	451 (29.1)		548 (35.0)		177 (11.1)	617 (38.7)	
Females	329 (44.6)		188 (29.3)		95 (13.7)	288 (41.6)	
Age Group				<0.001			0.012
< 50 Years	88 (30.8)		89 (31.2)		31 (10.3)	127 (42.5)	
50-70 Years	400 (29.4)		429 (33.4)		178 (13.0)	536 (39.1)	
> 70 Years	292 (45.3)		218 (31.8)		63 (10.2)	242 (39.3)	
Body Mass Index				0.030			0.54
≤ 30 kg/m^2	303 (33.1)		300 (32.9)		207 (11.7)	689 (39.2)	
>30 kg/m^2	477 (37.6)		436 (34.1)		65 (12.6)	216 (46.7)	
Diabetes Mellitus				<0.001			<0.001
Yes	194 (47.3)		110 (29.2)		202 (10.7)	747 (39.2)	
No	586 (31.3)		626 (34.0)		70 (17.4)	158 (39.7)	
Hypertension				<0.001			0.12
Yes	485 (36.7)		383 (34.2)		168 (12.6)	532 (39.4)	
No	295 (30.7)		353 (31.8)		104 (10.8)	374 (39.7)	
Estimated Glomerular Filtration Rate				<0.001			<0.001
< 60 mL/min/1.73m^2	121 (46.4)		78 (30.0)		25 (9.1)	80 (30.5)	
≥ 60 mL/min/1.73m^2	659 (32.4)		658 (33.9)		247 (12.7)	825 (41.1)	
C-reactive Protein				<0.001			0.029
≤ 5 mg/L	357 (28.0)		411 (34.4)		161 (12.5)	527 (40.9)	
> 5 mg/L	423 (41.9)		325 (31.7)		111 (11.0)	378 (37.9)	

Supplemental Table 3: Results of bootstrapping for the primary endpoint 25-hydroxyvitamin D using 1,000 samples with random replacement

Parameter	Bootstrapped Difference	BCa 95% CI	Standard Error	P-value
25(OH)D (ng/mL)				
Adequate vs. deficient/potentially deficient Mg status	0.38	0.62 to 1.24	0.47	0.51
Deficient Mg status vs. non-deficient Mg status	1.45	-0.03 to 2.92	0.75	0.06

Abbreviations: 25(OH)D, 25-hydroxyvitamin D; BCa, bias corrected accelerated; CI, confidence interval

Supplemental Table 4: Characteristics of unmatched and matched study participants with deficient/non-deficient or adequate serum Mg status

	Unmatched patients (n=2,286)			Matched patients (n=584)		
Parameter	Deficient Mg status n=295	Non-deficient Mg status n=1,991	SMD %	Deficient Mg status n=292	Non-deficient Mg status n=292	SMD%
Age (years)	62.3 ± 9.9	63.1 ± 10.7	7.8	62.3 ± 9.9	62.7 ± 10.4	3.9
Females	108 (36.6)	613 (30.8)	-17.5	108 (37.0)	104 (35.5)	-4.1
Body Mass Index (kg/m^2)	27.7 ± 4.2	27.4 ± 4.1	-7.2	27.7 ± 4.2	27.7 ± 4.3	0.0
Season of Blood Sampling						
Winter	128 (43.4)	429 (21.5)	-73.7	126 (43.2)	127 (43.5)	1.4
Spring	55 (18.6)	372 (18.7)	0.4	55 (18.8)	60 (17.1)	-6.2
Summer	64 (21.7)	586 (29.4)	23.5	64 (22.0)	65 (22.3)	1.3
Fall	48 (16.3)	604 (30.3)	-2.4	48 (16.4)	50 (17.1)	2.6
Smoking	178 (60.3)	1278 (64.2)	11.4	176 (60.3)	176 (60.1)	0.0
Exercise (scale)	5.8 ± 1.7	5.9 ± 1.8	5.7	5.8 ± 1.7	5.8 ± 1.8	0.0
eGFR (ml/min/1.73 m^2)	69 ± 16	65 ± 15	-25.8	69 ± 15	69 ± 15	0.0
Diagnosis						
Diabetes mellitus	74 (25.1)	340 (17.1)	-29.2	72 (24.7)	77 (26.3)	5.4
Hypertension	181 (64.4)	1141 (57.3)	-20.1	179 (61.3)	175 (59.7)	-4.0
Atrial Fibrillation	44 (14.9)	300 (15.1)	0.8	43 (14.7)	40 (13.7)	-4.0
Biochemical Parameters						
Bilirubin (mg/dL)	0.64 ± 0.38	0.64 ± 0.39	0.0	0.64 ± 0.38	0.66 ± 0.55	4.2
Sodium (mmol/L)	141 ± 3	141 ± 3	0.0	141 ± 3	141 ± 3	0.0
Calcium (mmol/L)	2.33 ± 0.11	2.33 ± 0.10	0.0	2.32 ± 0.10	2.32 ±0.11	0.0
Phosphate (mmol/L)	1.12 ± 0.17	1.14 ± 0.19	11.1	1.12 ± 0.17	1.11 ± 0.19	-5.5
PTH (pg/mL)	32 ± 16	35 ± 33	11.6	32 ± 16	32 ± 17	0.0
C-reactive protein (mg/dL)	0.87 ±1.75	0.99 ±1.95	6.5	0.87 ± 1.76	0.81 ± 1.44	-3.7
Cortisol (µg/dL)	22.2 ± 7.1	22.6 ± 8.5	5.1	22.1 ± 7.0	22.5 ± 6.5	5.9
Medications						
Beta-Blockers	180 (61.0)	1264 (63.5)	7.3	180 (61.6)	180 (61.4)	0.0
ACE-inhibitors/AT-blockers	172 (58.3)	1150 (57.8)	-1.4	172 (58.9)	173 (59.0)	0.9
Glucocorticoids	4 (1.4)	46 (2.3)	7.1	4 (1.4)	2 (0.7)	-7.6
Statins	128 (43.4)	957 (48.1)	13,2	128 (43.8)	124 (42.3)	-4.0
Digitoxin	53 (18.0)	295 (14.8)	-12.3	52 (17.8)	55 (18.8)	3.9
Diuretics	93 (31.5)	564 (28.3)	-9.9	93 (31.8)	92 (31.4)	0.9
Aspirin	203 (68.8)	1428 (71.7)	9.0	202 (69.2)	201 (68.6)	0.9
Antibiotics	3 (1.0)	36 (1.8)	6.8	3 (1.0)	4 (1.4)	3.7

Abbreviations: eGFR, estimated glomerular filtration rate; PTH, parathyroid hormone; ACE, angiotensin converting enzyme; AT, angiotensin; SMD, standardized mean difference
